# Cardioprotective drugs and heart failure/cardiomyopathy incidence in chemotherapy-treated cancer survivors of breast cancer and non-Hodgkin lymphoma: a retrospective cohort study in England

**DOI:** 10.1093/ehjopen/oeaf039

**Published:** 2025-04-25

**Authors:** Pooja Hindocha, Alexander R Lyon, Krishnan Bhaskaran, Helen Strongman

**Affiliations:** Department of Non-Communicable Disease Epidemiology, London School of Hygiene and Tropical Medicine, Keppel Street, London WC1A 7HT, UK; National Heart and Lung Institute, Imperial College London and Cardio-Oncology Centre of Excellence, Royal Brompton Hospital, Sydney Street, London SW3 6NP, UK; Department of Non-Communicable Disease Epidemiology, London School of Hygiene and Tropical Medicine, Keppel Street, London WC1A 7HT, UK; Department of Non-Communicable Disease Epidemiology, London School of Hygiene and Tropical Medicine, Keppel Street, London WC1A 7HT, UK

**Keywords:** Chemotherapy-induced cardiotoxicity, Cancer survivors, Electronic health records, Heart failure, Cardiomyopathy

## Abstract

**Aims:**

Evidence for the use of beta-blockers, angiotensin II receptor blockers (ARB), or angiotensin-converting enzyme inhibitors (ACEi) to mitigate chemotherapy-induced cardiotoxicity is inconclusive. The objectives are to investigate associations between prescription of ARBs, ACEis, and/or beta-blockers in the year following cancer diagnosis and subsequent risk of heart failure/cardiomyopathy (HF/CM) in chemotherapy-treated breast cancer and non-Hodgkin lymphoma (NHL) survivors.

**Methods and results:**

This cohort study used linked English electronic healthcare records from 9875 adult (≥18 years) breast cancer and NHL survivors who received chemotherapy. Cox regression was used to estimate the association between primary care-prescribed beta-blocker, ARB, and ACEi use in the year following cancer diagnosis, and subsequent HF/CM incidence, adjusting for potential confounders. Likelihood ratio tests were used to assess effect modification. The mean follow-up duration was 4.9 years (maximum 21.4). After adjusting for age, the risk of HF/CM was higher in the exposed group [hazard ratio (HR): 1.69, 95% confidence interval (CI): 1.34–2.14], but further adjustment for gender, comorbidities, and other medications reduced the association to close to null (HR: 1.07, 95% CI: 0.68–1.69). There was no evidence that the association differed by cancer site, age, radiotherapy, prior cardiovascular disease, or years since cancer diagnosis.

**Conclusion:**

We found no evidence that general practitioner prescribed beta-blocker, ARB, or ACEi use was associated with a reduced incidence of HF/CM in this population of chemotherapy-treated breast cancer and NHL survivors. This might be because the drug dosage and timing were not optimized to prevent chemotherapy-related cardiac damage; residual confounding by indication may also have obscured any treatment benefit.

## Structured Graphical abstract

**Graphical Abstract:**
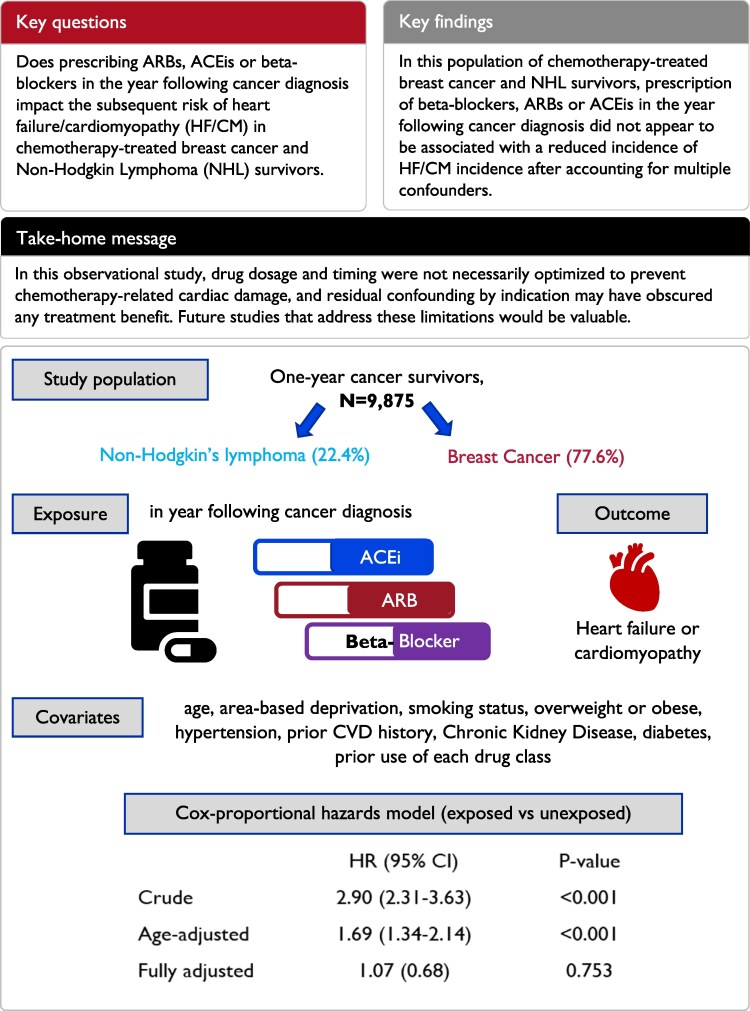

## Introduction

Cancer survival in the UK has doubled over the last 40 years.^1^ As survivors are living longer, they are faced by increasing risks of adverse cardiovascular events driven by the cardiotoxic effects of key cancer treatments such as chemotherapy, antibody treatment, and radiotherapy.^2,3^ Available literature suggests that a substantial proportion of chemotherapy-induced cardiotoxicity (CIC) is driven by anthracyclines^4,5^ and trastuzumab.^3^ A systematic review found that breast cancer and osteosarcoma patients receiving anthracyclines have a more than five-fold higher risk of clinical cardiotoxicity compared with those on non-anthracycline treatment.^4^ Another study found that compared with no chemotherapy, breast cancer patients treated with trastuzumab had a four-fold higher risk of heart failure/cardiomyopathy (HF/CM); the risk with trastuzumab and anthracycline combined was even higher.^6^

Dexrazoxane, an antioxidant agent, is currently the only EMA- and FDA-approved drug for treating anthracycline-induced cardiotoxicity, with a limited indication of use in women with metastatic breast cancer who have already received a doxorubicin dose of 300 mg/m^2^.^7,8^ There are, however, concerns that dexrazoxane leads to myelosuppression, secondary malignancies, and interference with the anti-tumour activity of anthracyclines,^7^ but the evidence is limited and conflicting.^9^ Given the increasing burden of CIC and limited approved treatment options, there has been an increasing interest in using beta-blockers, angiotensin II receptor blockers (ARBs), and angiotensin-converting enzyme inhibitors (ACEIs) to mitigate CIC. The 2005/2009 AHA Guidelines suggest that beta-blockers may help in the management of patients at a high risk of HF after chemotherapy.^10^ More recently, the European Society of Cardio-Oncology recommended the use of ARBs/ACEIs or beta-blockers in for both secondary prevention and treatment of cardiovascular disease (CVD) in line with general cardiology guidelines, and called for further evidence from Randomized Controlled Trials (RCT) designed to study cancer therapy-related cardiovascular toxicity.^11^ However, evidence for prophylactic use of these drugs is debated. Gujral *et al.*^12^ carried out a systematic review and meta-analysis to evaluate the impact of these drug classes on patients treated with anthracyclines with or without trastuzumab across several clinical trials and one observational study. Results showed that the use of ACEi/ARBs did not significantly affect mean left ventricular ejection fraction (LVEF) or HF diagnosis. Beta-blocker usage, on the other hand, was significantly associated with decreases in LVEF and a reduced risk of HF. Nevertheless, due to high heterogeneity of studies investigating this question, small sample sizes, and limited follow-up periods, evidence for the protective effect of ARBs, ACEis, and beta-blockers on CIC remains inadequate.

The goal of this study was to examine associations between cardioprotective drug use and cardiac outcomes in chemotherapy-treated patients using routinely collected primary care data in the UK, specifically investigating whether prescription of ARBs, ACEis, and/or beta-blockers in the year following cancer diagnosis was associated with the risk of HF/CM in chemotherapy-treated breast cancer and non-Hodgkin lymphoma (NHL) survivors.

## Methods

### Setting and data sources

This retrospective cohort study used primary care data collected from the Clinical Practice Research Datalink (CPRD GOLD) in England. The CPRD consists of anonymized patient records documented as part of routine care by general practitioners (GP) in the UK and Northern Ireland.^13^ Data collected include demographics, diagnoses, prescriptions, laboratory tests, and lifestyle information such as smoking and alcohol use. The patients in the database have been shown to be broadly representative of the UK population in terms of age, sex, and ethnicity.^13^ In this study, CPRD GOLD records were linked to: hospital admissions data from the Hospital Episode Statistics Admitted Patient Care database (HES APC); Index of Multiple deprivation (IMD), a small area-based measure of deprivation using the patient’s residence postcode;^14^ death registrations from the Office for National Statistics (ONS); and cancer registrations from the National Cancer Registration and Analysis service (NCRAS), for information on cancer diagnoses, severity, and treatment. Linkage of the databases limited the setting to England, with the study period spanning 1995–2017.

### Participants

**Figure 1:**
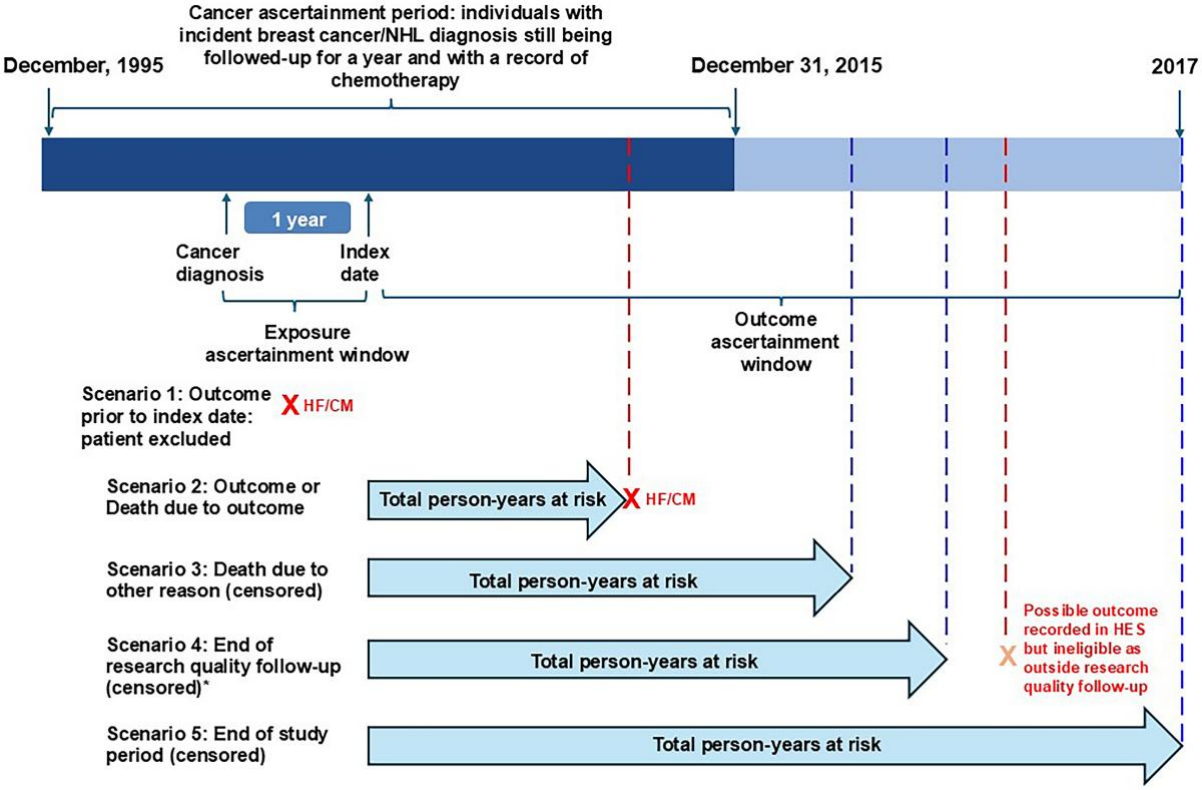
Study design overview, periods of recruitment, exposure, and outcome ascertainment. *Differs based on when research quality follow-up ends for those patients. NHL, non-Hodgkin’s lymphoma; HES, Hospital Episode Statistics; HF/CM, heart failure/cardiomyopathy.

Our development of cancer survivor cohorts has been described in a previous paper that used a broader cohort to describe medium- and long-term risks of specific cardiovascular diseases in survivors of 20 adult cancers.^15^ Briefly, we included individuals aged ≥18 years with incident breast cancer or NHL (identified by the first code of cancer at any of the linked databases^16^) during research quality follow-up in CPRD,^13^ who were alive and under follow-up for at least 1 year post-cancer diagnosis and had a record of chemotherapy in NCRAS in the year following diagnosis. We focused on breast cancer and NHL as these represent major cancer types that are commonly treated with anthracyclines and provided sufficient numbers for robust statistical analysis. Patients were excluded if they had <1 year of continuous research quality follow-up prior to cancer diagnosis (so that we could establish that included cancers were incident), history of other cancers or presence of secondary malignancies at the point of breast cancer/NHL diagnosis, no matching tumour record in the cancer registry (as corresponding cancer treatment details for these individuals are missing), or any record of HF/CM prior to study entry. The index date was the 1-year anniversary of cancer diagnosis, and patients were followed up until the earliest occurrence of outcome/death due to outcome, death due to cause other than outcome, end of research quality follow-up, or end of study period; any patient with a record for the outcome prior to the index date was excluded (*Figure 1*).

### Exposures, outcomes, and covariates

Patients were considered exposed if their primary care record showed at least two prescriptions of beta-blockers, ARBs, and/or ACEis in the year following cancer diagnosis. The outcome was incident diagnosis of HF/CM (composite outcome), defined using Read codes in CPRD GOLD derived from the CVD focused CALIBER project,^17^ International Classification of Diseases, version 10 (ICD-10) codes in HES, or ICD-9 and -10 codes for HF/CM as a cause of death in ONS mortality data.^16^ This composite outcome measure was chosen because codes for HF and CM appear to have been used interchangeably in the recording of a common pathophysiology relating to anthracycline toxicity in the clinical setting.

Covariates considered in the analysis were time-updated attained age (5 year categories with the first and last being larger due to the lower number of observations: 18–38, 78-maximum); sex; IMD quintile; body mass index (BMI) coded into categories using WHO criteria,^18^ i.e. underweight (<18.5), healthy (18.5–<25), overweight (25–<30), obese (≥30); smoking status (non-, current, or ex-smoker); and alcohol use categories by levels of drinking behaviour and as a binary problem drinking variable. The presence of other comorbidities known to be associated with the outcome and indications for the exposure were also included. These comorbidities were diabetes, hypertension, chronic kidney disease (CKD), migraine status, and other CVD, all defined using primary care Read codes. Prior use (at least two prescriptions in a year before cancer diagnosis) of ARBs, beta-blockers, ACEis, statins, and non-steroidal anti-inflammatory drugs (NSAIDs), along with the current use (at least two prescriptions in a year following cancer diagnosis) of statins and NSAIDs were also considered. Note that prior exposure to ARBs, ACEis, and beta-blockers was coded both as a combined variable (prior use of at least one drug type) to align with how the main exposure variable is categorized, and individually (three separate variables) in a sensitivity analysis. Lastly, tumour-specific and cancer treatment variables such as stage, grade, and receipt of radiotherapy were collected. A causal framework of assumed relationships between exposure, outcome, and several (measured and unmeasured) covariates can be found in Supplementary material online, *Figure S1*. Code lists for all study variables can be found online.^16^

### Statistical analysis

Descriptive statistics for the distribution of the covariates and total person-time at risk within the cohort were calculated. Cox proportional hazards regression methods using the index date as the origin were used to obtain the unadjusted hazard ratio (HR) for the association between the use of cardioprotective drugs and HF/CM, accounting for time in follow-up. The competing risk of death without the outcome was handled by modelling the cause-specific hazard, operationalized by censoring such deaths; this is appropriate when the question of interest is causal.^19^

The association between exposure and outcome was then adjusted for time-updated attained age during follow-up. In order to control for confounding without compromising power, the following modelling strategy was applied:^20^ (i) all potential confounders with complete data were included in the unadjusted model (Model 1), (ii) variables with a small proportion of missing data (up to 2% of participants) were included in a complete records analysis (Model 2), (iii) variables with a higher proportion of missing data (up to 20%) were only included in the model if they changed the estimated HR by 5%; the reference models for comparison excluded observations missing these data, (iv) variables with ≥20% missingness (namely cancer grade and stage) were not included in our initial approach due to significant loss of power, but in a *post hoc* sensitivity analysis, we added these variables using a multiple imputation approach (chained equations approach with 10 imputations using ordinal logistic regression with all variables from the substantive outcome model). Unless otherwise specified, all hypothesis tests were carried out using Wald tests. We produced curves to show the absolute cumulative incidence of HF/CM in the exposed and unexposed groups; to adjust for confounding in these curves, we standardized the curves to the covariate distribution of the exposed group, by fitting a flexible parametric survival model with the same parameters as our final Cox model, and predicting from this model cumulative incidence in the exposed group, with exposure set first to 1 and then to 0.

We used likelihood ratio tests comparing models with and without interaction terms to test whether the observed associations varied by the following factors: age at diagnosis, cancer site, receipt of radiotherapy, CVD history prior to index date, calendar time, and years since diagnosis. Stratified HRs were also estimated.

To investigate the association of beta-blockers separately with the incidence of HF/CM, in a *post hoc* analysis, the exposure variable was modified to distinguish between unexposed patients, patients who are exposed to cardioprotective drugs *including* beta blockers, and patients who are exposed to cardioprotective drugs excluding beta-blockers. This modified exposure replaced the main exposure variable in the final model and adjusted HRs were estimated. We investigated the impact of using a composite HF/CM through a sensitivity analysis repeating the final model with HF as the outcome.

All data manipulation and analyses were carried out using Stata (StataCorp LP, Version 16).^21^

This study was approved by the London School of Hygiene and Tropical Medicine Ethics Committee (approval 21888) and the Independent Scientific Advisory Committee for the Medicines and Healthcare Products Regulatory Agency Database Research (approval 16_274A2). Clinical Practice Research Datalink has approval to supply pseudonymized data for public health research without individual patient consent.^22^

## Results

**Figure 2:**
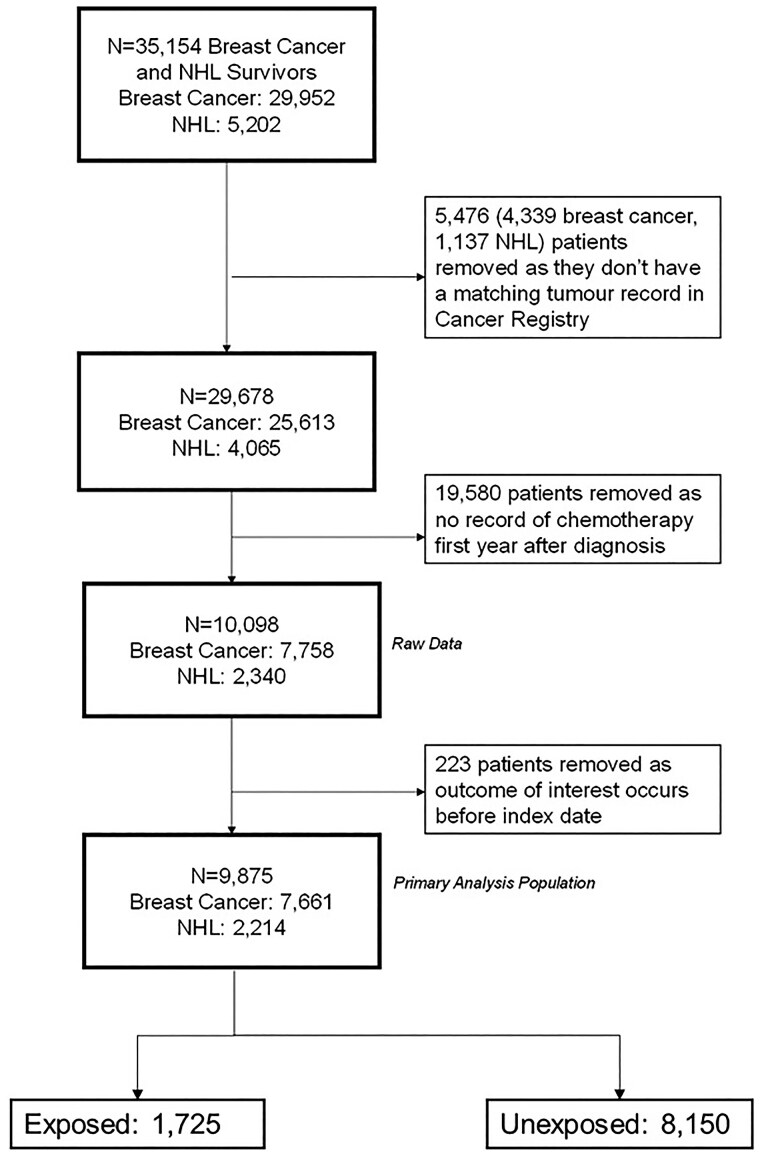
Study population profile. Flow diagram showing how the primary analysis population was derived. *N*, total number of participants; NHL, non-Hodgkin’s lymphoma; Index date, 1-year anniversary of cancer diagnosis.

**Figure 3:**
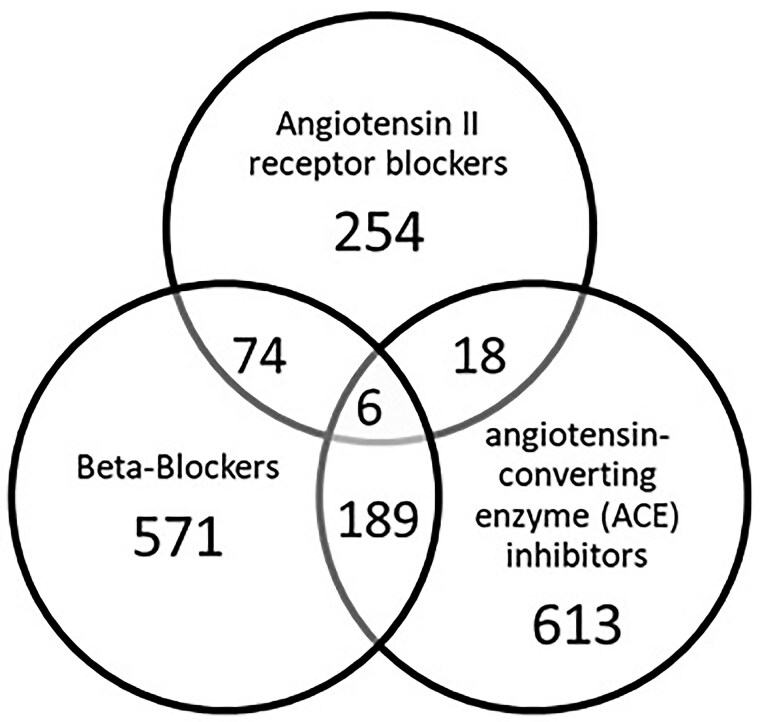
Distribution of drug classes within the exposure group (*N* = 1725).

The final analysis cohort consisted of 9875 patients (*Figure 2*). Of these, 1725 (17.5%) were exposed to at least one of beta-blockers, ARBs, or ACEis and 287/1725 (16.6%) were on medications that fit in two or more drug class categories (*Figure 3*).

A little over three-quarters of the cohort (7661/9875, 77.6%) were breast cancer survivors and 22.4% (2214/9875) were NHL survivors (*Table 1*). Breast cancer survivors were predominantly in the 18–59 years age group (5558/7661, 72.6%), while NHL survivors were predominantly in the older age group (≥60 years; 1372/2214, 62.0%). The exposed group was older at baseline than the unexposed group [mean age (SD) 63.1 (10.9) and 53.6 (11.9), respectively]. Compared with the unexposed group, a higher proportion of exposed patients were in the most deprived IMD category, were current and ex-smokers, or were obese. The exposed group also had a higher proportion of patients with other comorbidities (diabetes, CKD, hypertension, and history of other CVD) and with prescriptions for one or more cardioprotective drug classes in the year prior to (statins, beta-blockers, ACEis, and ARBs) or following cancer diagnosis (statins).

**Table 1:** Characteristics of study population by the exposure group

Variables	Distribution in sample	Prescription of ARBs, ACEi, or beta-blockers in year following cancer diagnosis		No prescription	
	*N* = 9875	*N* = 1725		*N* = 8150	
	*n* (%)	*n*	% (column)	*n*	% (column)
Socio-demographic characteristics					
Sex					
Male	1203 (12.2)	313	18.1	890	10.9
Female	8672 (87.8)	1412	81.9	7260	89.1
Age at cancer diagnosis^a^					
18–59	6400 (64.8)	645	37.4	5755	70.6
≥60	3475 (35.2)	1080	62.6	2395	29.4
Index of multiple deprivation (patient level)					
1 (least deprived)	2652 (26.9)	423	24.5	2229	27.3
2	2387 (24.2)	400	23.2	1987	24.4
3	2046 (20.7)	362	21.0	1684	20.7
4	1591 (16.1)	287	16.6	1304	16.0
5 (most deprived)	∼1 200^b^ (∼12.2)	253	14.7	∼940^b^	∼11.6
Missing	≤5^b^ (≤0.05)	0	0.0	≤5^b^	≤0.06
Lifestyle/behavioural					
Smoking status					
Non-smoker	4986 (50.5)	794	46.0	4192	51.4
Current smoker	1819 (18.4)	258	15.0	1561	19.2
Ex-smoker	2666 (27)	633	36.7	2033	24.9
Missing	404 (4.1)	40	2.3	364	4.5
Alcohol status and level					
Non-drinker	967 (9.8)	192	11.1	775	9.5
Ex-drinker	600 (6.1)	165	9.6	435	5.3
Current drinker (light)	5933 (60.1)	1007	58.4	4926	60.4
Current drinker (moderate)	527 (5.3)	108	6.3	419	5.1
Current drinker (heavy)	124 (1.3)	18	1.0	106	1.3
Current drinker (unknown)	520 (5.3)	111	6.4	409	5.0
Missing	1204 (12.2)	124	7.2	1080	13.3
Heavy drinking (current or previous)					
No	9562 (96.8)	1677	97.2	7885	96.7
Yes	313 (3.2)	48	2.8	265	3.3
Baseline health characteristics					
Body mass index categories					
Underweight (<18.5)	110 (1.1)	6	0.3	104	1.3
Healthy weight (≥18.5 to <25)	3710 (37.6)	415	24.1	3295	40.4
Overweight (≥25 to <30)	2948 (29.9)	566	32.8	2382	29.2
Obese (≥30)	2060 (20.9)	638	37.0	1422	17.4
Missing	1047 (10.6)	100	5.8	947	11.6
Hypertension at diagnosis					
No (low/not measured)	8457 (85.6)	1138	66.0	7319	89.8
Yes	1418 (14.4)	587	34.0	831	10.2
Record of cardiovascular disease prior to index date					
No	8008 (81.1)	1060	61.4	6948	85.3
Yes	1867 (18.9)	665	38.6	1202	14.7
Chronic kidney disease (≥Stage 3 at diagnosis)					
No	9311 (94.3)	1455	84.3	7856	96.4
Yes	564 (5.7)	270	15.7	294	3.6
Diabetes at diagnosis					
No	9378 (95.0)	1429	82.8	7949	97.5
Yes (Type 1 or Type 2)	497 (5.0)	296	17.2	201	2.5
Migraine status at diagnosis					
No	8953 (90.7)	1554	90.1	7399	90.8
Yes	922 (9.3)	171	9.9	751	9.2
Medication use before or in year after diagnosis					
ACEi use prior to diagnosis					
No	9055 (91.7)	981	56.9	8074	99.1
Yes	820 (8.3)	744	43.1	76	0.9
Beta-blockers use prior to diagnosis					
No	9058 (91.7)	1006	58.3	8052	98.8
Yes	817 (8.3)	719	41.7	98	1.2
ARB use prior to diagnosis					
No	9538 (96.6)	1410	81.7	8128	99.7
Yes	337 (3.4)	315	18.3	22	0.3
Statin use prior to diagnosis					
No	8816 (89.3)	1093	63.4	7723	94.8
Yes	1059 (10.7)	632	36.6	427	5.2
Statin use in year following diagnosis					
No	8818 (89.3)	1082	62.7	7736	94.9
Yes	1057 (10.7)	643	37.3	414	5.1
NSAID use prior to diagnosis					
No	8892 (90.1)	1494	86.6	7398	90.8
Yes	983 (10.0)	231	13.4	752	9.2
NSAID use in year following diagnosis					
No	8822 (89.3)	1498	86.8	7324	89.9
Yes	1053 (10.7)	227	13.2	826	10.1
Cancer treatment–specific data					
Cancer site					
Breast cancer	7661 (77.6)	1167	67.7	6494	79.7
NHL	2214 (22.4)	558	32.3	1656	20.3
Radiotherapy					
No	3869 (39.2)	770	44.6	3099	38.0
Yes	6006 (60.8)	955	55.4	5051	62.0
Tumour grade					
1	588 (6.0)	93	5.4	495	6.1
2	2923 (29.6)	442	25.6	2481	30.4
3	4214 (42.7)	684	39.7	3530	43.3
4	26 (0.3)	8	0.5	18	0.2
Missing	2124 (21.5)	498	28.9	1626	20.0
Tumour stage					
1	994 (10.1)	159	9.2	835	10.2
2	2373 (24.0)	344	19.9	2029	24.9
3	690 (7.0)	142	8.2	548	6.7
4	516 (5.2)	126	7.3	390	4.8
Missing	5302 (53.7)	954	55.3	4348	53.4

ACEis, angiotensin-converting enzyme inhibitors; ARBs, angiotensin II receptor blockers; NSAIDs, non-steroidal anti-inflammatory drugs.

^a^5558/7661 (72.6%) of breast cancer survivors were aged 18–59 years, 2103/7661 (27.4%) were aged 60+ years, 842/2214 (38.0%) of NHL survivors were aged 18–59 years, and 1372/2214 (62.0%) were aged 60+ years.

^b^Numbers in the missing category are suppressed to avoid small cell counts. Numbers in the most deprived category are rounded to the nearest 10 to avoid disclosing the suppressed cell counts by subtraction.

**Figure 4:**
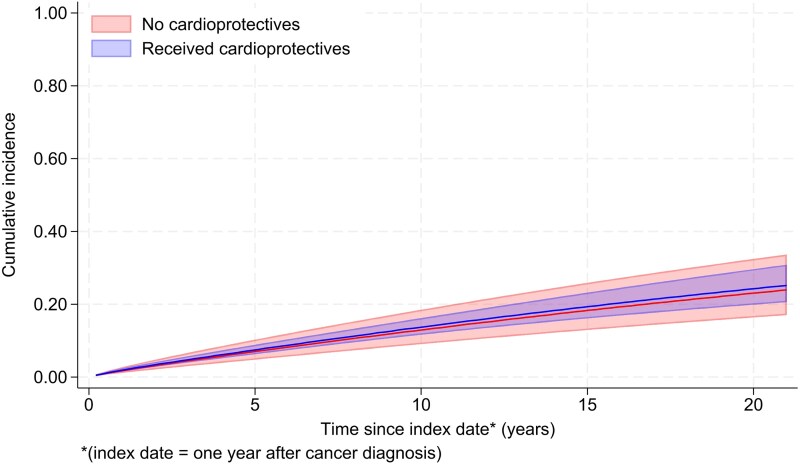
Cumulative incidence of heart failure/cardiomyopathy in people receiving and not receiving cardioprotective drugs, standardized to the covariate distribution of the former group.

During the 48 515 total person-years at risk (PYAR; mean follow-up: 4.9 years, maximum: 21.4), 3.5% of the patients had a record of HF/CM (345/9875). The remaining patients were censored due to end of follow-up (*n* = 7360, 74.5%) or death without the outcome (*n* = 2,170, 22.0%) The absolute incidence of HF/CM was 16.2 per 1000 PYAR [95% confidence interval (CI): 13.5–19.5; 155 events/7097 PYAR] in the exposed group and 5.6 per 1000 PYAR (95% CI: 4.9–6.3; 230 events/41419 PYAR) in the unexposed group (unadjusted HR accounting for time since the index date = 2.90, 95% CI: 2.31–3.63, *P* < 0.001) (*Table 2*). The HR was substantially reduced after adjusting for time-updated attained age during follow-up [age-adjusted HR: 1.69 (95% CI: 1.34–2.14, *P* < 0.001)]. After adjusting for all confounders with complete data, the HR decreased further to 1.11 (95% CI: 0.71–1.74) with no evidence that the observed association was more than expected by chance (*P* = 0.64). The inclusion of IMD in a complete records analysis had a minimal impact on the HR [HR: 1.07 (95% CI: 0.68–1.69, *P* = 0.75)]. The final adjusted model presented in *Table 2* included the combined variable for prior use of ARBs/ACEIs and beta-blockers. *Figure 4* shows the estimated absolute cumulative incidence of HF/CM in the exposed and unexposed groups, standardized to the covariate distribution of the former to adjust for confounding. A sensitivity analysis with adjustment for individual variables resulted in an HR of 1.30 (95% CI: 0.89–1.91). The inclusion of smoking status or BMI did not change the HR by ≥5%; these variables were not included in the final model. Stage and grade were missing for more than 20% of the cohort and hence were also not included in the final model, but in a sensitivity analysis using multiple imputation, the HR adjusted for these variables was very similar to our main model (HR 1.06, 95% CI: 0.67–1.68).

**Table 2:** Multivariable Cox proportional hazards analysis: hazard ratios for heart failure/cardiomyopathy comparing the exposed and unexposed groups

Model type	*N*	Number of events	Person-years at risk	HR	95% CI	*P*-values*
Unadjusted	9875	345	48 515	2.90	2.31–3.63	<0.001
Adjusted for age	9875	345	48 515	1.69	1.34–2.14	<0.001
M1	9875	345	48 515	1.11	0.71–1.74	0.641
M2	9871	345	48 500	1.07	0.68–1.69	0.753

All models adjusted for time in follow-up, i.e. origin is the index date.

M1—inclusion of all variables that have complete data, i.e. exposure, age attained, gender, hypertension, cardiovascular disease prior to the index date, alcohol use (heavy vs. non-heavy), chronic kidney disease, diabetes, migraine, prior use of ACEi/beta-blockers/ARBs (combined), prior use of statins/NSAIDs, use of statins/NSAIDs in year following treatment, and radiotherapy.

M2—inclusion of variables missing 1–2% of the data (complete records analysis), i.e. all variables in M1 and IMD.

CI, confidence intervals; HR, hazard ratio; IMD, index of multiple deprivation; M, model.

^*^Wald *P*-values of association between exposure and outcome.

The results of the *post hoc* analysis to delineate associations with the use of beta-blockers separately (without ARBs/ACEIs) suggested no evidence of association between the use of beta-blockers and HF/CM risk (HR 1.12, 95% CI: 0.69–1.80, *P* = 0.64). Repeating the main model with an HF only outcome gave results consistent with the main analysis (HR 1.06, 95% CI: 0.68–1.67).

**Figure 5:**
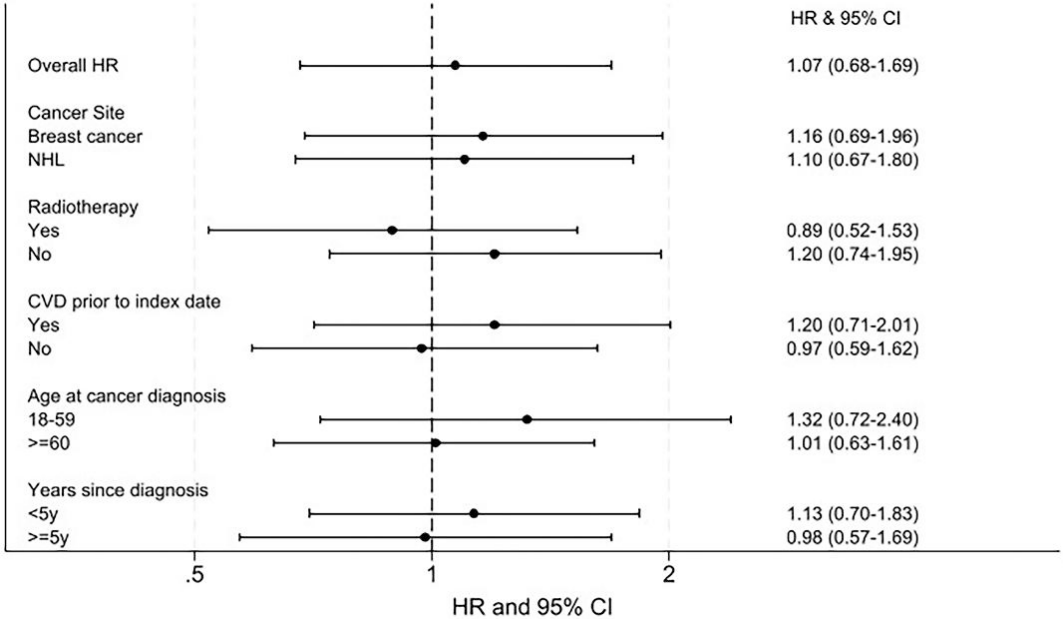
Overall and stratified association between cardioprotective drugs and heart failure/cardiomyopathy. Likelihood ratio tests comparing models with and without the interaction terms were used to examine if there was any evidence against the null hypothesis of no interaction. There was no statistical evidence that cancer site, age at diagnosis, radiotherapy, prior cardiovascular disease, or years since cancer diagnosis modified the relationship between exposure and outcome. CI, confidence intervals; CVD, cardiovascular disease; HF/CM, heart failure/cardiomyopathy; HR, hazard ratio, NHL, non-Hodgkin’s lymphoma.

Considering interaction by the pre-specified variables, after adjusting for confounding, there was no evidence that the relationship between exposure and outcome differed according to cancer site, radiotherapy, history of CVD, age at cancer diagnosis, or (time-updated) time since cancer diagnosis (*Figure 5*). A *post hoc* analysis adjusting for calendar time (1994–99, 2000–04, 2005–09, 2010–14), with and without an interaction term, provided no evidence that calendar time confounded the association (no change in HR) or changed over time (calendar year-adjusted HR 1.06, 95% CI: 0.68–1.67, *P*-value for interaction with calendar time 0.17). Hazard ratios stratified by calendar time and provided in Supplementary material online, *Table S1*.

## Discussion

### Key findings

In this population, the prescription of ARBs, ACEIs, and/or beta-blockers was not associated with the incidence of HF/CM (*Structured Graphical Abstract*). The crude incidence of HF/CM was approximately three times higher for individuals prescribed to beta-blockers, ARBs, and/or ACEIs compared with those who were not (HR: 2.90, 95% CI: 2.31–3.63), reflecting the older age distribution and higher prevalence of risk factors in the exposed group. After adjusting for measured confounders, the strength of association was close to null (HR: 1.07, 95% CI: 0.68–1.69) with wide CI indicating a high degree of uncertainty. Stratified analyses provided no statistical evidence that the association between the prescription of ARBs, ACEIs, and/or beta-blockers and HF/CM incidence differed by cancer site, age at cancer diagnosis, radiotherapy, prior CVD disease, or years since cancer diagnosis.

### Results in context

Most of the other research exploring this topic has been through clinical trials, and while the statistical evidence for the impact of these drugs classes on mitigating CIC remains ambiguous, the point estimates for published effect measures almost universally suggest a protective effect for these drugs,^12,23–25^ suggesting that they may have some efficacy when given in a protocolled and targeted way to mitigate chemotherapy-related cardiotoxicities. Our study more likely captured routine incidental use around the time of cancer treatment but not necessarily targeted at or optimized for preventing chemotherapy-related problems.

Another observational study which investigated the effect of beta-blocker use in chemotherapy-treated breast cancer patients^26^ analysed 318 patients with a median follow-up of 3.2 years. This small single-centre US study did find strong evidence that continuous beta-blocker use was associated with an 80% lower rate of HF incidence. Based on the results of our *post hoc* analysis modifying the exposure variable to specifically examine associations with beta-blocker usage, there was no corresponding evidence of association between the use of beta-blockers and incidence of HF/CM in our data. A key difference in the US study was that the authors defined beta-blocker exposure as confirmed continuous use throughout chemotherapy treatment; we were unable to replicate this due to data limitations (as discussed below). An additional factor is that the Fine and Gray model results from the study are not directly comparable to our results because the differences they estimated in the cumulative incidence of HF could have been driven by competing risks (e.g. the exposed group having a lower cumulative incidence of HF due to higher risk of cancer death). Finally, the different setting and different range of variables available for analysis (e.g. including ethnicity, baseline LVEF) could have further contributed to the discrepant results.

### Strengths and limitations

Availability of electronic health records databases such as CPRD allow for studies to be carried out in a time- and cost-effective way with more patients and longer follow-up than what would be feasible in a randomized clinical trial. The present study is, to our knowledge, the largest to date addressing this research question. The maximum follow-up in clinical trials that explored similar questions was ∼2.6 years^27^; in the present analysis, the average follow-up for the whole cohort was almost double that, at 4.9 years, with some patients followed for substantially longer; although this shows considerable heterogeneity in terms of length of follow-up, our use of time-to-event methods and censoring meant this was appropriately handled from a statistical perspective. Additionally, by linking CPRD GOLD with other databases, we were able to accurately identify cardiovascular outcomes^28,29^ including HF and critical covariates such as IMD (as a proxy for socioeconomic status) and cancer treatment variables, could be obtained.

There are, however, important limitations. Despite the large base population of CPRD GOLD, the relatively low rate of outcome events in our study population meant that our precision (as expressed in our CI) was not sufficient to rule out some treatment benefit, which could have been missed due to sampling variation. As with any retrospective studies using electronic healthcare records, the data used in this study were not collected for research purposes. Some potentially important variables such as family history of CVD, ethnicity, cardioprotective drug dose, and radiotherapy field and doses were not available for analysis. We also lacked detailed information on chemotherapy and other systemic anti-cancer treatments, including exact drugs and dosages received; if individuals in the exposed group were subjected to more cardiotoxic chemotherapy regimens (e.g. including anthracyclines/trastuzumab), any benefit of cardioprotective treatment may have been masked. Furthermore, although anthracyclines are a mainstay of chemotherapy treatment in breast cancer and NHL, the lack of detailed anti-cancer treatment data meant we could not be sure that every individual in our study population was anthracycline-exposed—this may have led to dilution of any real benefit of cardiovascular medication on anthracycline-related cardiotoxicity in our results. Unmeasured confounding may also have arisen if comorbidities or other relevant medications were not perfectly captured, especially given the higher proportion of comorbidities observed in the (older) exposed group. Depending on the extent of the difference, confounding by indication may make an effective treatment seem ineffective or even harmful.^30^ High missingness of tumour stage and grade meant these variables were not adjusted for in our primary analysis, though a sensitivity analysis using the available data gave little indication of confounding.

There are also key considerations to note with respect to the exposure. We did not know the intent of treatment for the cardioprotective drugs, and prescriptions were identified through primary care rather than cardiologist records. Some patients were already receiving these medications before cancer diagnosis, while others received them shortly after cancer diagnosis—potentially in response to early indications of cardiac dysfunction. This heterogeneity in prescribing patterns and intent makes it difficult to isolate any preventive effect. Additionally, any benefit of cardioprotective drugs could be sensitive to timing of use;^24,31^ if treatment was not aligned with chemotherapy exposure, the window of potential benefit could have been missed.^3^ It is also important to note that our study specifically examined whether pre-existing or early prescription of these medications was associated with reduced incidence of subsequent HF/CM; a different design would be needed to address the separate question of whether these medications are beneficial once established cardiac dysfunction develops. The exposure definition was based on at least two prescription records in the patient’s primary care file, not including prescriptions made in hospitals or drugs purchased over the counter as this information is not available.^32^ It is hence possible that some exposed individuals were incorrectly classified as unexposed *if* they obtained any of the drugs from a non-GP setting, leading to non-differential misclassification of exposure and biasing the effect estimate towards the null. Additionally, an assumption made with regard to the exposure was that prescription equates to individuals taking the treatment as intended. Perfect adherence to the treatment regimen is unlikely. If patients in the exposed group did not take the drugs as prescribed, any real treatment effect would be diluted.

As mentioned previously, this study had limited power to disentangle the individual effects of specific drug classes, and this is made more difficult by the fact that some patients are prescribed to multiple drugs. While we attempted to investigate the impact of beta-blockers individually, nearly a third of these patients were also prescribed to at least one of ARBs or ACEIs. We also did not have detail on individual drugs within a class to understand whether there were differential effects for products specifically tested for cardio-protection in this setting.

Our primary outcome definition included both HF and CM. This reflected the clinical experience that these terms have tended to be used interchangeably to capture the broad concept that the recent European Society of Cardiology guidelines term ‘cancer treatment related cardiac dysfunction’.^11^ We thus made an implicit assumption of a common pathophysiology, but it is a limitation of the coded data available that we cannot be sure that these outcomes were truly homogeneous in this respect. A sensitivity analysis using only codes for HF gave results consistent with the main analysis. We did not have echochardiogram results or data on cardiac biomarkers such as brain natriuretic peptide (BNP) available in these routine healthcare records to further explore the clinical presentation of HF, or to look at CM subtypes. Lastly, the study had limited power to investigate variation in effect between patients with different characteristics (interactions); whilst we noted no evidence of effect modification, these analyses would need to be repeated with larger sample sizes to draw more definitive conclusions about how the adjusted HR differs between subgroups. The use of propensity score methods might also have improved the power of the study, but we decided to use a multivariable regression approach, to more easily show the effect of including different levels of confounder control, and incorporating variables with missing data; propensity score methods would have a similar ability to control for confounding as our approach.^33^

## Conclusions

Overall, our analysis found no evidence of an association that GP-prescribed beta-blockers, ARBs, or ACEis were associated with a reduced incidence of HF/CM in this population of chemotherapy-treated breast cancer and NHL survivors, though we note that heterogeneity in prescribing patterns and intent made it difficult to isolate any preventive effect. We cannot rule out that a real benefit of these treatments was missed due to limited statistical power, residual confounding, or because drug dosage and timing was not optimized to prevent chemotherapy-related cardiac damage. Future studies addressing these limitations and considering other potentially cardioprotective therapies such as statins would be valuable.

## Supplementary Material

oeaf039_Supplementary_Data

## Data Availability

Our licence to use these data prevents us from sharing individual patient data with third parties. The raw data may be requested directly from the CPRD following their usual procedures.

## Lead author biography

Pooja Hindocha, MSc, completed this project as a part of her MSc in Epidemiology at the London School of Hygiene and Tropical Medicine, supervised by Dr Helen Strongman. Prior to this, she worked as a Research Assistant and Project Manager within the Bioinformatics Department at EpiVax, Inc., a biotech company in RI (USA) focusing on vaccine development and therapeutic risk assessment. She now works in industry and is interested in exploring the fields of non-communicable disease epidemiology, particularly within the pharmaceutical sector, with a focus on leveraging real-world evidence to inform clinical and public health decisions.
