# Supplementary material for: Cardioprotective drugs and heart failure/cardiomyopathy incidence in chemotherapy-treated cancer survivors of breast cancer and non-Hodgkin lymphoma: a retrospective cohort study in England
Source: Eur Heart J Open. 2025 Apr 25;5(3):oeaf039. doi: 10.1093/ehjopen/oeaf039 (PMC12066945; doi:10.1093/ehjopen/oeaf039)
Supplement: oeaf039_Supplementary_Data [file oeaf039_supplementary_data.zip › Supplementary Material.docx]

**Supplementary Material**

**Supplemental Figure 1**: Causal Framework showing assumed relationships between exposure, outcome, and covariates

**Supplemental table 1: Results of supplementary analysis including categorical calendar year as an independent variable, with and without an interaction term**

| **Model** | **outcome events** | **Person-years of follow-up** | **HR (95% CI)** |
| --- | --- | --- | --- |
| Original adjusted model | 345 | 48515 | 1.07 (0.68, 1.69) |
| Categorical calendar year added as an independent variable | 345 | 48515 | 1.06 (0.68, 1.67) |
| Categorical calendar year added as an interaction term* |  |  |  |
| 1994 to 1999 | 42 | 6232 | 0.92 (0.36, 2.32) |
| 2000 to 2004 | 101 | 16040 | 1.47 (0.83, 2.60) |
| 2005 to 2009 | 134 | 17465 | 0.81 (0.48, 1.38) |
| 2010 to 2014 | 68 | 8779 | 1.24 (0.67, 2.30) |

*p-value for interaction estimated using a Likelihood Ratio Test = 0.172
