# Supplementary figures and images for: Cardioprotective drugs and heart failure/cardiomyopathy incidence in chemotherapy-treated cancer survivors of breast cancer and non-Hodgkin lymphoma: a retrospective cohort study in England
Source: Eur Heart J Open. 2025 Apr 25;5(3):oeaf039. doi: 10.1093/ehjopen/oeaf039 (PMC12066945; doi:10.1093/ehjopen/oeaf039)

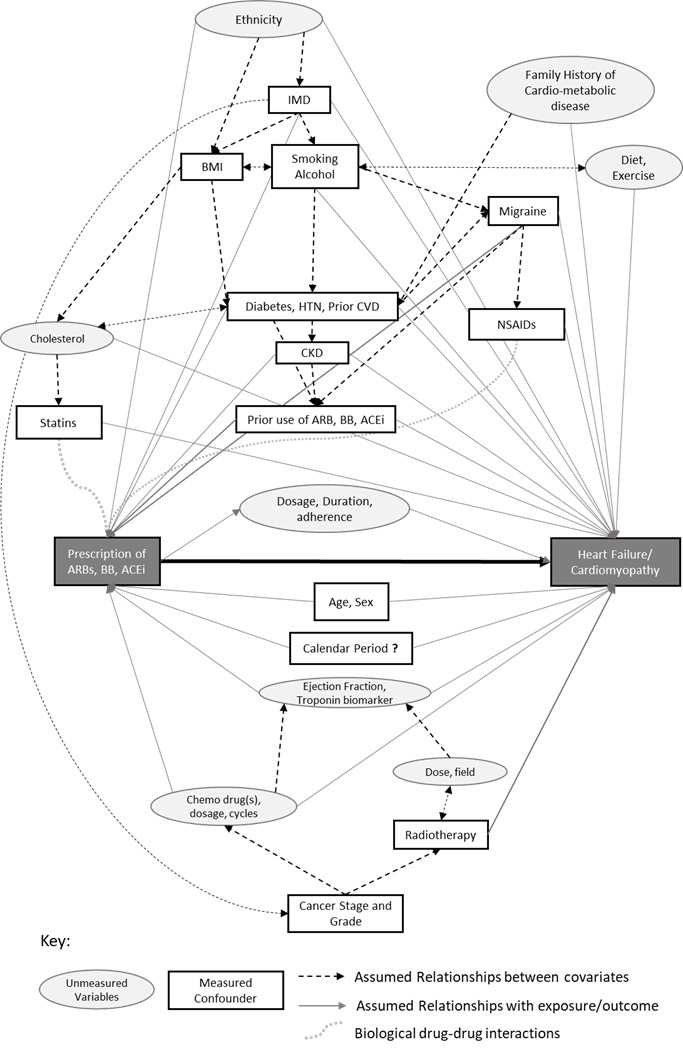

Supplement: oeaf039_Supplementary_Data [file oeaf039_supplementary_data.zip › EHJ-QCCO Supp Figure 1.jpg]
